# Peer Attachment and Academic Procrastination in Chinese College Students: A Moderated Mediation Model of Future Time Perspective and Grit

**DOI:** 10.3389/fpsyg.2019.02645

**Published:** 2019-11-26

**Authors:** Hexiang Jin, Wenchao Wang, Xiaoyu Lan

**Affiliations:** ^1^Student Mental Health Education and Counseling Center, Northwest Minzu University, Lanzhou, China; ^2^Faculty of Psychology, Beijing Normal University, Beijing, China

**Keywords:** academic procrastination, peer attachment, future time perspective, grit, college students

## Abstract

Although prior research has considerably documented the prevalence and correlates of academic procrastination in college students, relatively little is known about the role of longer volitional processes of goal striving, such as grit, on academic procrastination; moreover, the knowledge about direct and interactive effects of social context and personal characteristic on facilitating grit, which in turn mitigate academic procrastination, are still underexplored. Given these gaps in the existing literature, the current study, more exploratory in nature, investigates a moderated mediation model of future time perspective and grit in the association between peer attachment and academic procrastination in Chinese college students. A total of 1,098 undergraduate students (43.2% girls) aged from 18 to 25 were involved in the current study, and participants were asked to fill in a battery of self-report questionnaires. Results indicated that (a) peer attachment was negatively and significantly associated with academic procrastination; (b) grit partially mediated the association between peer attachment and academic procrastination; more precisely, peer attachment was positively associated with grit, which in turn was negatively linked to academic procrastination; and (c) future time perspective moderated the association between peer attachment and grit; more specifically, for students with low levels of future time perspective, the association between peer attachment and grit turned out to be significantly negative. These findings suggest that interventions targeting the enhancement of peer attachment and grit may prevent or reduce academic procrastination, and college students who regard future orientation as pessimistic should be paid specific attention by university-based counseling services.

## Introduction

Academic procrastination, defining as an intended delay of study-related action despite expecting to be worse off for the delay (Steel, 2007), has sparked the research interests of social scientists in the last decades. Based on the previous report (e.g., Steel, 2007), a total of 75–90% undergraduate students is estimated to delay completing academic tasks. Given its prevalence among undergraduate students, further investigation of exploring the correlates of academic procrastination and the potential ways to reduce academic procrastination is assumed to be potentially valuable.

According to prior research, academic procrastination poses a serious threat to students’ academic achievement and subjective well-being (Kim and Seo, 2015; Steel and Klingsieck, 2016). Along with these negative effects, in the last decades, an escalating body of research has documented the correlates of academic procrastination among undergraduate students in different cultural contexts (Steel, 2007; Zhang et al., 2018; Chen, 2019), leading to a more nuanced understanding of this phenomenon. According to these research efforts, the majority of research has claimed that self-regulation failure is one of the main causes of procrastination (e.g., Steel, 2007; Steel and Klingsieck, 2016). Despite these empirical findings, relatively little is known about the role of longer volitional processes of goal striving, such as grit, on academic procrastination. In a similar vein, exploring the factors that can facilitate grit, which in turn reduce the level of academic procrastination in undergraduate students, is assumed to be imminent especially in the context of Chinese culture. For instance, academic performance and school success are highly emphasized because of longstanding cultural linkage between academic success and family dignity (Quach et al., 2015; Lan et al., 2019d). As such, college students who fail to achieve better academic performance are more likely to encounter additional stress from sociocultural and parental expectations. Given this significance, further investigation into the correlates of longer volitional processes of goal striving and related factors is valuable in terms of university-based counseling services with research-based suggestions for interventions.

To fill these gaps, we use the ecological framework, as an overarching ground, to further address the correlates of academic procrastination (Bronfenbrenner, 1979). As documented by this framework, individuals are embedded within layers of environmental systems, and adaptive development (e.g., academic performance) unfolds through the dynamic interactions between the individual and multiple contexts. This framework has been successfully applied to explore the ecological correlates of academic procrastination in Chinese individuals (Chen and Han, 2017). Although the investigation of the multiple interactions on academic procrastination is potentially informative, academic procrastination is a complex process that involves various contextual and individual influences. Therefore, it is difficult to encompass all possible correlates into one simple moderation model (i.e., only take interaction effects into consideration). We then resort to several related theories (i.e., attachment theory, temporal motivation theory, and socioemotional selectivity theory; see the literature review below) to gain a more comprehensive understanding of the correlates of academic procrastination. Based on an exploratory approach, we propose a moderated mediation model (i.e., the pattern of mediation varies as a function of the moderator) of future time perspective and grit in the association between peer attachment and academic procrastination in Chinese college students. In the following sections, we conduct a literature review to map out the possible associations of peer attachment, future time perspective, and grit with academic procrastination.

### Peer Attachment and Academic Procrastination

According to the attachment theory (Bowlby, 1977; Bucci et al., 2015), individuals who experience secure attachment experiences are more likely to have goal orientation, and are motivated to use effective coping mechanisms for solving challenges. By contrast, individuals who do not have a sufficiently secure base are more likely to regard challenging situations as stressful and use ineffective methods, such as procrastination (Krause and Freund, 2014; Yildiz and Iskender, 2019). In this regard, the quality of attachment relationships may be highly associated with academic procrastination.

Although traditional attachment theory asserts that first attachment relationships are established with parents, individuals often form enduring attachment bonds in subsequent moments of the life course outside of their family (e.g., peers; Gorrese and Ruggieri, 2012). Moreover, college students often encounter a critical period of learning and personal growth, and they are not prone to obeying the strict discipline imposed by their parents and teachers at school, making self-discipline as a crucial element for their success. As such, many of these students find themselves facing personal and academic challenges independently for the first time in their lives (Geng et al., 2018). Moreover, during this period, individuals expand their network and establish mature relationships with peers, and above all with romantic partners, as these relationships become progressively more central in their daily life (Guarnieri et al., 2015). Recently, a growing literature has shown that peers as attachment figures are influential sources of social and emotional support (e.g., Badenes-Ribera et al., 2019; Lan and Radin, 2019b). In this perspective, peer attachment, as the central arena of adult attachment relationships, may become an effective social resource to ameliorate academic procrastination.

Peer attachment refers to the affectional bonding in peers, including trust, reliance, as well as sharing personal thoughts and emotions (Bowlby, 1977; Armsden and Greenberg, 1987). Although, up to our knowledge, there is no single investigation exploring the association between peer attachment and academic procrastination in undergraduate students, some of the existing findings may provide possible indications. For example, research shows the positive association between peer attachment and academic adjustment in college students (Swenson et al., 2008), suggesting that peer attachment may be negatively associated with academic procrastination. In terms of Chinese college students, peer attachment is found to be negatively associated with several problem behaviors, such as Internet addiction (Yang et al., 2016) and depressive symptoms (Ying et al., 2007). On the basis of these findings, we propose that peer attachment is negatively and directly associated with academic procrastination in college students.

Furthermore, apart from the direct association between peer attachment and academic procrastination, the underlying mechanism between them is still unclear in the existing literature. Given this knowledge gap, we propose that grit may serve as a candidate mediator between peer attachment and academic procrastination in college students. The theoretical and empirical rationales of the mediating role of grit in this association are addressed in the following section.

### The Mediating Role of Grit

Temporal motivation theory (Steel, 2007) proposes that several poor self-regulation tendencies, including self-regulation traits, which could impinge on the nature of the association between social context and procrastination. Among self-regulation traits, we propose that grit is more appropriate to fulfill our research purpose. Grit involves perseverance and passion for long-term goals, especially in the face of obstacles and adversities (Duckworth et al., 2007). To date, research has consistently shown the positive role of grit in facilitating academic functioning (Wolters and Hussain, 2015; Credé et al., 2017; Lam and Zhou, 2019), such as better academic performance and less tendencies to be academically procrastinated. For example, Wolters and Hussain (2015) found that grit is negatively related to academic procrastination in undergraduate students. In terms of collective cultural contexts, a similar pattern has been replicated (e.g., Datu et al., 2016; Lee, 2017). For instance, Lee (2017) documented that grit is negatively associated with global psychological stress experienced by college students. This may indicate that gritty students are more capable of dealing with stress, which in turn hinders the tendency to be procrastinated. Indeed, achieving challenging goals, such as better academic performance, requires the willingness to control impulses and work hard, as well as the ability to manage challenges associated with goal pursuit (Ivcevic and Brackett, 2014). Based on these findings, we assume that grit is negatively associated with academic procrastination in undergraduate students.

Furthermore, self-regulation occurs in a rich social context in which other individuals play a profound role in guiding individual action (Orehek et al., 2017). From a theoretical perspective, attachment theory is an especially useful ground for understanding the link between social context and self-regulation (Bowlby, 1977; Armsden and Greenberg, 1987). An important role of the attachment figure is to serve as a secure base from which individuals strive toward personal goals. This may indicate that attachment orientations influence how individuals engage in self-regulation. Prior research has shown that attempts to improve or alter the course of self-regulation should consider the role of interpersonal processes, such as peers (Orehek et al., 2017). From an empirical perspective, research concerning the antecedents of grit shows that the relatedness to friends is positivity associated with grit in students (Datu, 2017); similarly, Lan (2019) found that peer attachment is positively associated with grit in Chinese college students, indicating that positive peer relationships can facilitate the levels of grit.

Despite aforementioned findings, how attachment orientations and grit directly and indirectly influence academic procrastination in undergraduate students is still less investigated in the existing literature. In response to the fractionated studies on the associations of peer attachment and grit with academic procrastination, we aim to examine these direct and indirect associations in one single investigation. Additionally, given the obvious importance of time to procrastination, time orientation seems to be a particularly essential individual factor to consider in terms of self-regulation and academic procrastination (Ferrari and Díaz-Morales, 2007). The theoretical and empirical rationales of the role of time orientation in the pathways of these associations are elucidated in the following section.

### The Moderating Role of Future Time Perspective

As documented by the socioemotional selectivity theory (Carstensen et al., 1999), the perception of time plays a fundamental role in the selection and pursuit of goals. When time is perceived as expansive and optimistic, focusing on long-term goals is prioritized, otherwise achieving short-term goals becomes more important (Lang and Carstensen, 2002). Indeed, students who have committed to a purpose or direction are more likely to report higher levels of grit than those who lack a sense of direction in life (Hill et al., 2016). Furthermore, Zimbardo and Boyd (1999) consider orientations to the past, the present and the future as a basic dimension of human functioning. In the current study, we focus on future time perspective, because an emphasis on future time orientation focusing on a long-time perspective is in line with the nature of grit (i.e., long-term goals). Moreover, the importance of a future time perspective has been acknowledged in educational psychology concerning desired educational outcomes, such as academic engagement (Horstmanshof and Zimitat, 2007).

Future time perspective is defined as the capacity to foresee, anticipate, and plan for future desired outcomes (Zimbardo and Boyd, 1999). Prior research has shown that future time perspective is beneficial to well-being, academic engagement, and adaptive behavior (Horstmanshof and Zimitat, 2007; Kooij et al., 2018). Moreover, study shows that having a life direction can predict grit in undergraduate students (Hill et al., 2016). According to these indications, we assume that future time perspective may have an impact on academic procrastination through the role of grit. The specific associations of these variables are informed by socioemotional selectivity theory (Carstensen et al., 1999), which posits that the interaction between individual perceptions of the future and the quality of personal networks affects individuals’ goal orientation. Likewise, the empirical finding has provided evidence for the interdependence between time perspective and the quality of social relationships that affects the degree to which we cope with stress (Holman, 2015). Although the roles of peer attachment and future time perspective are potentially interchangeable from a statistical standpoint^1^, we regard peer attachment as an independent variable and future time perspective as a moderator in relation to grit due to the following theoretical and empirical perspectives. First, we are interested in understanding how and when peer attachment may influence academic procrastination in undergraduate students because of the knowledge gap in the existing attachment literature (see the literature review under the section of peer attachment and academic procrastination). Second, the direct influence of time orientation on academic procrastination has been relatively well-documented in the literature (Ferrari and Díaz-Morales, 2007; Sirois, 2014) than do the role of peer attachment; in the meantime, the knowledge of the multiple effects of future time perspective and social context on academic procrastination is relatively underexplored. On the basis of such findings and considerations, we propose that high levels of future time perspective may enhance the positive association between peer attachment and grit; in turn, high levels of grit are negatively associated with academic procrastination in college students.

### The Present Study

**FIGURE 1:**
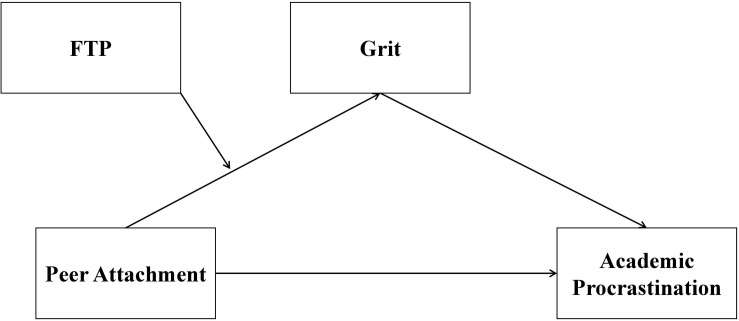
A hypothesized moderated mediation model. Age, gender, and socioeconomic status were considered as control variables. FTP, future time perspective.

In sum, the goals of the current study are twofold: (a) to explore the direct and indirect effects of peer attachment and grit on academic procrastination of Chinese undergraduate students, and (b) to ascertain whether future time perspective can moderate the association between peer attachment and grit. Moreover, as indicated by prior research (Steel, 2007; Steel and Klingsieck, 2016) – the potential association between sociodemographic variables and academic procrastination – we regard age, gender, and socioeconomic status (SES) as potential covariates in the further course of analysis. Specifically, we propose a moderated mediation model (see Figure 1) to examine the following hypotheses (H):

H1. Peer attachment is negatively associated with academic procrastination (H1a); grit mediates the association between peer attachment and academic procrastination; more precisely, peer attachment is positively associated with grit, which in turn is negatively related to academic procrastination in undergraduate students (H1b).
H2. Future time perspective moderates the association between peer attachment and grit. Given the lack of studies on the moderating role of future time perspective in the peer attachment-grit link, an exploratory hypothesis is generated in this regard. To be specific, higher levels of future time perspective may enable college students who possess secure peer attachment to report higher levels of grit, as compared with those reporting lower levels of future time perspective.

## Materials and Methods

### Participants and Procedures

Prior to data collection, ethical approval for the current study was obtained at Northwest Minzu University. Through personal networks (i.e., based on convenience samples), the authors contacted several public colleges in Lanzhou, China (Lanzhou is the capital city of Gansu Province, which is located in Northwest mainland China). The public universities are usually entitled to recruit students from all provinces in mainland China based on a certain proportion. This renders us an opportunity to recruit participants who come from different parts of China, which may lessen the potential influence of intercultural differences on the outcome of this study. After a long process negotiation, the principals from four public universities finally agreed to collaborate with us in this project. These universities were Northwest Minzu University, Lanzhou University of Technology, Lanzhou University of Finance and Economics, and Gansu Agricultural University. Data collection was administrated from October 2017 to September 2019^2^, with the assistance of trained research assistants and psychology teachers. Each of the potential respondents was asked to use their mobile phone to scan the QR code, which was shown on the blackboard through an electronic projector during the public health classes. At the beginning of this survey, the research purposes and participants’ rights (e.g., confidentiality, anonymity, non-coercion, and right to withdraw) were explained to potential respondents, and they were asked to fill in a battery of self-report questionnaires online through Wenjuanwang. Only on the condition that participants agreed with these pieces of information would the follow-up survey start. Overall, the participation rate was approximately 95%, which is in line with prior research of Chinese students (Lan et al., 2019c; Lan and Wang, 2019c). Suggested by prior research (Lan et al., 2019e), inserting missing values and skipping the questions would prompt a reminder to double-check before submission. This was done to avoid missing out any questions, but participants were also informed that, if they felt uncomfortable or wanted to skip any specific question, they should feel free to leave it blank or withdraw. Moreover, to ensure the authenticity of the data, a portable electronic device (i.e., mobile phone) was only allowed to scan the QR code once. In addition, the duration of completing this survey was recorded automatically, and the relatively short completion time would prompt a further check when conducting the further course of data analysis. These procedures were all set up based on the online system prior to data collection.

Based on convenience samples, a total of 1,098 Chinese undergraduate students aged from 18 to 25 years (*M*_age_ = 20.33, *SD* = 1.67; 43.2% girls) were involved in the current study. Of these participants, 479 (43.6%) undergraduates were in their first year; 247 (22.5%) were in their second year; 175 (15.9%) were in their third year; and 195 (17.9%) were in their fourth year. Concerning parental education background, 57.2 and 68.4% of fathers and mothers have completed middle school education or lower; 30.0 and 22.3% have completed high school education; 11.7 and 8.7% have completed undergraduate education; and 1.1 and 0.5% have completed postgraduate education or higher, respectively.

### Measures

#### Sociodemographic Characteristics

Participants were asked to complete the sociodemographic information on their year of birth and gender (self-identified as: 1 = male, 2 = female) in the process of this survey. Moreover, SES was assessed via the maternal and paternal educational levels. Although prior research has suggested that a composite score encompassing parental education level, parental occupation, and family income, would be better to capture the “full picture” of family SES (e.g., Lan and Zhang, 2019d), the current study regards parental education level only as the indicator of family SES due to the following theoretical and methodological considerations. First, the outcome of this study focuses on the academic domain. As suggested by prior research (Spera et al., 2009), parental education level is significantly and positively related to higher parental aspirations, which in turn may potentially influence the academic functioning of their children. Second, as documented by prior research (Currie et al., 2008), data on family SES is difficult to collect from young people, because they are not willing to reveal these pieces of information, such as parental occupation and family income. This often results in low completion or high non-response rates. To minimize the potential risk of non-response rates, we only select parental education level as the indicator of family SES in this study. As suggested by prior research (Lan et al., 2019a), we provided four choices for the participants: (a) middle school graduation or lower, (b) high school graduation, (c) bachelor’s degree graduation, and (d) master’s degree graduation or higher. Both paternal and maternal educational levels were summarized to yield a composite score, with higher values indicating greater family SES.

#### Academic Procrastination

Academic procrastination was measured by the 19-item Aitken Procrastination Inventory (API; Aitken, 1982). This scale has been validated in Chinese individuals by Chen et al. (2008). One of the examples is “I delay starting things so long I do not get them done by the deadline.” Participants rated each item on a 5-point scale ranging from 1 (*completely disagree*) to 5 (*completely agree*). All the items were averaged to yield a composite score, with higher levels of score indicating the severity of procrastination toward academia. Prior research has shown good internal consistency of this scale (Chen et al., 2008). In the current study, Cronbach’s alpha was 0.79.

#### Peer Attachment

Peer attachment was assessed using the 25-item Inventory of Peer Attachment (IPPA; (Armsden and Greenberg, 1987). IPPA has been validated in Chinese populations by Zhang et al. (2011). One of the examples is “My friends accept me as I am.” Participants were asked to assess the quality of their relationships with peers on a 5-point Likert-type ranging from 1 (*almost never or never true*) to 5 (*almost always or always true*). All the items related to alienation were reversed, and the average score was yielded to represent peer attachment. Thus, higher values indicated secure peer attachment. Previous research has demonstrated good internal consistency of this scale in Chinese populations (Ying et al., 2007; Lan and Radin, 2019b). Cronbach’s alpha was 0.91 in the current study.

#### Grit

Grit was measured by the 8-item Grit Scale (Duckworth and Quinn, 2009). This scale has been validated in Chinese populations by Li et al. (2018). One of the examples is “New ideas and projects sometimes distract me from previous ones.” Participants were asked to rate each item from 1 (*not like me at all*) to 5 (*very much like me*) on the Likert scale. The average score of eight items was calculated, with a higher score indicating higher levels of grit. Previous studies have shown good internal consistency of this scale in Chinese populations (Lan, 2019; Lan et al., 2019b). Cronbach’s alpha was 0.75 in the present study.

#### Future Time Perspective

Future time perspective was measured by a Chinese version of the Future Time Perspective Scale invented by Song (2004). This scale contains 20 items, and one of the examples is that “I think my future is rosy.” Participants were asked to rate each item from 1 (*completely disagree*) to 5 (*completely agree*) on the Likert scale. The average of 20 items was calculated, with a higher score indicating the greater capacity to perceive future time as expansive and optimistic. Previous study has shown good internal consistency of this scale in Chinese college students (Fan et al., 2016). Cronbach’s alpha was 0.89 in the present study.

#### Analytic Plan

Data analyses were performed using SPSS 21.0 (IBM Corporation, 2012). First, we conducted analyses of descriptive statistics and zero-order correlation to have a preliminary overview of study variables. Second, to explore the first and second hypotheses, we conducted data analyses in two steps. These procedures were suggested by prior research (i.e., Hipson et al., 2019). At an initial step, we computed the mediation analysis to estimate the direct and indirect effects of peer attachment and grit on academic procrastination. If direct and indirect effects were significant, we then explored a moderated mediation model to estimate the change of the indirect effect as a function of future time perspective. Based on the theoretical perspective (Carstensen et al., 1999), we examined future time perspective as a moderator in the pathway linking peer attachment to grit only. We used the PROCESS macro for SPSS (version 2.16.3; Hayes, 2013) to run the mediation and moderated mediation analyses, using models 4 and 7, respectively. Direct and indirect effects were estimated using Preacher and Hayes’ bias-corrected non-parametric bootstrapping techniques with 5000 bootstrap samples (Preacher and Hayes, 2004). Suggested by prior research (Settanni et al., 2018), the existence of mediation and moderated mediation effects were further evaluated using 95% bias-corrected CIs. If the confidence intervals did not contain zero, these effects were considered statistically significant.

## Results

### Preliminary Analyses

Means and standard deviations for study variables and bivariate correlations are reported in Table 1.

**TABLE 1:** Descriptive statistics and intercorrelations between study variables.

**Variables**	***M***	***SD***	**Range**	**1**	**2**	**3**	**4**	**5**	**6**	**7**
1. Peer attachment	3.55	0.55	1–5	–						
2. Grit	3.02	0.65	1–5	0.07^∗^	–					
3. Future time perspective	3.34	0.62	1–5	0.35^∗∗∗^	0.49^∗∗∗^	–				
4. Academic procrastination	2.54	0.51	1–5	–0.30^∗∗∗^	–0.51^∗∗∗^	–0.54^∗∗∗^	–			
5. Age	20.33	1.67	18–25	–0.02	–0.22^∗∗∗^	–0.22^∗∗∗^	0.22^∗∗∗^	–		
6. Gender ^a^	–	–	1–2	0.14^∗∗∗^	–0.11^∗∗∗^	–0.13^∗∗∗^	0.04	0.30^∗∗∗^	–	
7. Socioeconomic status	2.98	1.27	2–8	0.10^∗∗∗^	–0.13^∗∗∗^	–0.03	0.07^∗^	0.03	0.11^∗∗∗^	–

**N = 1,098. ^*a*^Coded as 1, male; 2, female.*^∗^*p* < 0.05, ^∗∗∗^*p* < 0.001.*

As shown in Table 1, peer attachment was positively associated with grit and future time perspective, and negatively associated with academic procrastination; grit was positively associated with future time perspective, and negatively associated with academic procrastination; future time perspective was negatively associated with academic procrastination. In terms of covariates, age was negatively associated with grit and future time perspective, and positively associated with academic procrastination; gender was positively linked to peer attachment, and negatively associated with future time perspective and academic procrastination; SES was positively associated with peer attachment and academic procrastination, and negatively linked to grit.

### Testing for a Moderated Mediation Model

**FIGURE 2:**
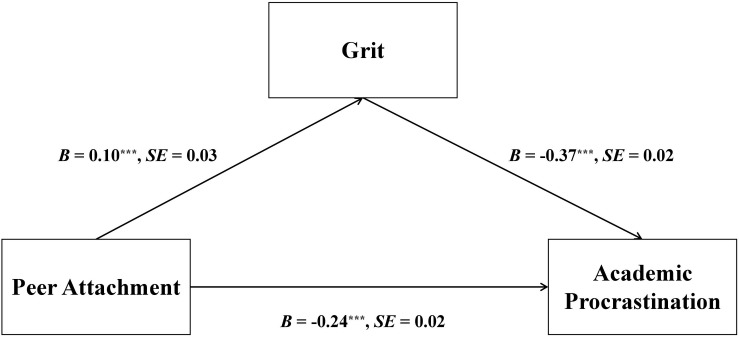
Unstandardized path coefficients depicting mediation of grit in the association between peer attachment and academic procrastination. ^∗∗∗^*p* < 0.001.

**FIGURE 3:**
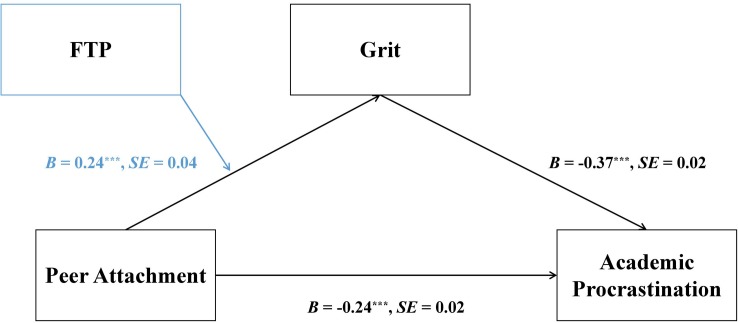
Unstandardized path coefficients depicting a moderated mediation role of future time perspective and grit in the association between peer attachment and academic procrastination. ^∗∗∗^*p* < 0.001.

After controlling for age, gender, and SES, there was a significant mediation effect found in the prediction of academic procrastination, *B* = −0.04, *SE* = 0.02, *p* < 0.001, 95% CI [−0.07, −0.01], whereby peer attachment was positively associated with grit, which in turn was negatively associated with academic procrastination (see Figure 2). Further analysis revealed a significant moderated mediation in the pathway linking peer attachment to academic procrastination, *B* = −0.09, *SE* = 0.02, *p* < 0.001, 95% CI [−0.13, −0.05] (see Figure 3).

Path coefficients of the moderated mediation model are reported in Table 2. As shown in Table 2, after controlling for age, gender, and SES, peer attachment and future time perspective were negatively associated with grit^3^, and the interaction between peer attachment and future time perspective was positively linked to grit. In addition, grit and peer attachment were each negatively associated with academic procrastination.

**TABLE 2:** Moderated mediation model analysis.

**Outcome variables**	**Independent variables**	***B***	***SE***	***t***	***p***	**95% CI**
Grit	Constant	5.63	0.59	9.54	< 0.001	[4.47, 6.79]
	Age	–0.04	0.01	–4.50	< 0.001	[−0.06, −0.02]
	Gender^a^	0.02	0.03	0.81	0.41	[−0.04, 0.09]
	SES	–0.05	0.01	–4.24	< 0.001	[−0.08, −0.03]
	Peer attachment	–0.91	0.15	–5.98	< 0.001	[−1.21, −0.61]
	FTP	–0.35	0.16	–2.18	0.02	[−67, −0.03]
	Peer attachment x FTP	0.24	0.04	5.40	< 0.001	[0.15, 0.33]
	*R*^2^	0.29				
	*F*	76.79^∗∗∗^				
Academic procrastination	Constant	3.82	0.20	19.03	< 0.001	[3.42, 4.21]
	Age	0.03	0.01	4.32	< 0.001	[0.01, 0.05]
	Gender^a^	–0.01	0.02	–0.47	0.63	[−0.06, 0.04]
	SES	0.01	0.01	1.35	0.17	[−0.01, 0.03]
	Grit	–0.37	0.02	–18.23	< 0.001	[−0.41, −0.33]
	Peer attachment	–0.24	0.02	–10.76	< 0.001	[−0.29, −0.20]
	*R*^2^	0.34				
	*F*	116.24^∗∗∗^				

**N = 1,098. ^*a*^Coded as 1, male; 2, female; SES, socioeconomic status; FTP, future time perspective.*^∗∗∗^*p* < 0.001.*

Furthermore, as shown in Table 3, the bias-correct centile bootstrap method showed that the direct effect of peer attachment on academic procrastination was −0.24, and that the index of moderated mediation was −0.09. Both the direct and indirect effects did not include zero in terms of 95% confidence intervals. That is, grit partially mediated the association between peer attachment and academic procrastination, and future time perspective moderated the link between peer attachment and grit.

**TABLE 3:** Direct and indirect effects of study variables.

**Variables**	***B***	***SE***	**95% CI**	
**Direct effect of peer attachment on academic procrastination**				
	–0.24	0.02	–0.29	–0.20
**Conditional indirect effects peer attachment on academic procrastination via Grit at levels of future time perspective**				
*M* – 1 *SD* (2.71)	0.09	0.01	0.05	0.13
*M* (3.34)	0.03	0.01	0.01	0.06
*M* + 1 *SD* (3.97)	–0.01	0.01	–0.05	0.02
**Index of moderated mediation**				
	–0.09	0.02	–0.13	–0.05

**N = 1,098. Bootstrap sample size = 5,000. LL, low limit; 95%CI, 95% confidence interval; UL, upper limit.**

**FIGURE 4:**
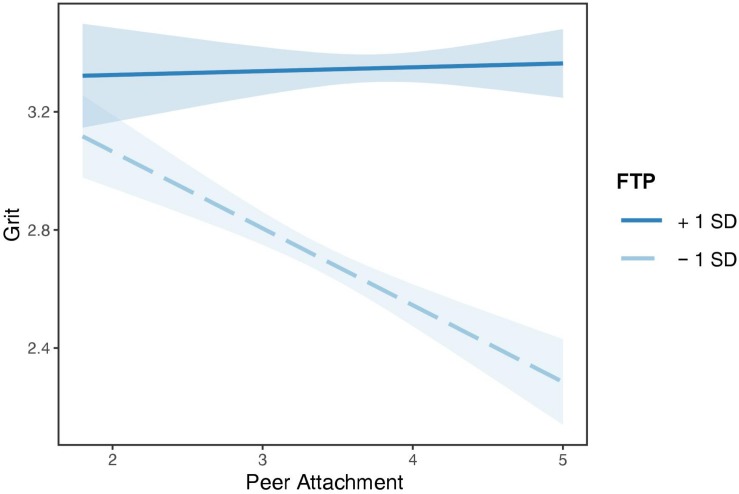
Interaction effect of peer attachment and future time perspective on grit. Future time perspective was divided into two levels based on mean: low = *M* − 1 *SD*, high = *M* + 1 *SD*. Corresponding bands represent 95% confidence intervals. FTP, future time perspective.

The nature of the significant interaction effect was examined by a simple slope analysis. As shown in Figure 4, future time perspective was divided into two levels based on mean (low = *M* − 1 *SD*, high = *M* + 1 *SD*). The association between peer attachment and grit was significant only for individuals with lower levels of future time perspective (*B* = −0.26, *SE* = 0.04, *t* = −6.07, *p* < 0.001), but not for those with higher levels of future time perspective (*B* = 0.01, *SE* = 0.04, *t* = 0.30, *p* = 0.77).

## Discussion

Although prior research has considerably documented the prevalence and correlates of academic procrastination in college students, relatively little is known about the role of longer volitional processes of goal striving, such as grit, on academic procrastination; moreover, the knowledge about direct and interactive effects of social context and personal characteristic on facilitating grit, which in turn mitigate academic procrastination, are still underexplored. Given these gaps in the existing literature, the goal of this study, more exploratory in nature, is to investigate the associations of peer attachment, future time perspective, and grit with academic procrastination in Chinese college students. Our findings showed that grit partially mediated the negative association between peer attachment and academic procrastination. Moreover, future time perspective moderated the association between peer attachment and grit. Particularly, the significant and negative association between peer attachment and grit was found for students who possessed lower levels of future time perspective only.

Our first purpose was to examine the direct and indirect associations of peer attachment and grit on academic procrastination. The findings showed that peer attachment was negatively and directly associated with academic procrastination. Such a finding is in line with the attachment theory (Bowlby, 1977) and previous research of Chinese college students (Ying et al., 2007; Yang et al., 2016), suggesting that college students who establish positive peer relationships and hold a secure attachment orientation are more motivated to use effective coping mechanisms for solving academic challenges. Indeed, during the period of undergraduate education, peer relations are highly emphasized, because students regard self-discipline and independence as crucial elements for their success (Swenson et al., 2008). In this context, a secure peer attachment may inspire college students to cope with difficulties more efficiently and complete academic assignments on time. Particularly, this impact may be more heightened in Chinese culture. In such a context, extrafamilial relations are highly underlined (Huang, 2012), and integration in peer groups can facilitate their adaptive competencies, such as academic functioning.

Moreover, in line with the first hypothesis, the current findings supported that grit mediated the association between peer attachment and academic procrastination; to be specific, peer attachment was positively associated with grit, which in turn was negatively associated with academic procrastination. Such a finding is compatible with prior research indicating that peer attachment is positively associated with grit (Lan, 2019), and that grit is a positive facilitator of academic functioning (Wolters and Hussain, 2015; Lam and Zhou, 2019). The current study brings all these fractionated studies together, demonstrating the pathway from peer attachment to academic procrastination via grit. One possible explanation relies on the claim that attachment orientations influence how individuals engage in self-regulation (Orehek et al., 2017). Positive peer relationships, served as a secure base, may inspire individuals to strive toward goal orientations. With this respect, gritty students are more capable of dealing with academic difficulties, and are more likely to express increased confidence and to complete academic tasks on time.

Our second objective was to ascertain whether future time perspective may moderate the association between peer attachment and grit. The current findings showed that the association between peer attachment and grit was not always significant, which was dependent on the levels of future time perspective. Such a finding confirms the socioemotional selective theory (Carstensen et al., 1999), suggesting that when regarding future time as pessimistic, individuals may prioritize short-term goals, which can impede the levels of grit. Although secure peer attachment is assumed to be a catalyst for positive traits, such as grit, prior research has also documented the “risk” role of peer relationships on problem behaviors. For example, research shows that the more adolescents are identified with the high-risk group, the more aggressiveness they show (Pokhrel et al., 2010). This may indicate that the more adolescents are affiliated with the potential high-risk group characterized by pessimistic time orientation, the less gritty they are. Nevertheless, for those with high levels of future time perspective, they often regard themselves as gritty. This is in line with prior research suggesting that having a life direction can predict grit in undergraduate students (Hill et al., 2016). In such a case, the role of social support, such as secure peer attachment, may be independent of the levels of grit. This is because future time perspective is considered as a personal asset (Seginer, 2008), wherein individuals often mobilize this asset to overcome adversity; that is, in the context of insecure peer attachment, future time perspective may compensate this vulnerability, and enable individuals to be gritty. Although such an explanation is plausible, further investigation is still needed to confirm these associations given the nature of this preliminary evidence.

While the current study adds to the existing literature by documenting the associations of peer attachment, future time perspective, and grit with academic procrastination in Chinese college students, some limitations should be acknowledged when interpreting the current findings. First, the cross-sectional and correlational design does not allow to infer the causation of the study variables. For example, whether there is a reciprocal relationship between peer attachment and grit should be ascertained in the further course of research. Second, future time perspective is a multidimensional construct, and previous research has shown that components of future time perspective impact individual traits diversely, such as hope and resilience (e.g., Davis and Cernas Ortiz, 2017). In a similar vein, this study does not distinguish the two facets of grit; however, several studies has indicated the distinct associations of perseverance and consistency with peer attachment in different developmental periods (e.g., Lan, 2019), and distinct associations of perseverance and consistency with academic performance and problem behaviors (e.g., Chen et al., 2018; Weisskirch, 2018; Lan and Radin, 2019b). Thus, future studies should unpack the dimensions of future time perspective and grit or use a person-centered approach to address the associations among study variables (e.g., Ma et al., 2019). Third, this study is limited by a single methodology approach, which is potentially influenced by social desirability or common method bias (Podsakoff et al., 2003). For instance, participants cannot evaluate themselves accurately and may not report honestly in terms of procrastination behaviors. Therefore, future study should utilize a multi-informant and/or mixed-methods approach to address these associations involved in this study. Fourth, due to several constraints, the duration of data collection process of this study is relatively long, which may potentially influence the reliability of the current findings. Thus, caution should be kept in mind when interpreting these results, and further course of research should avoid this issue. Fifth, despite some theoretical and empirical considerations, the narrow operationalization of SES in this study deserves further attention. Future initiatives should consider encompassing traditional indicators of family SES (i.e., parental education level, parental occupation, and family income; see Lan and Zhang, 2019d) to confirm the current findings. Finally, this study relies on a monocultural dataset, which delimits the possibility to generalize the findings into different cultural contexts. Prospective future investigation should consider conducting a cross-cultural design to ascertain the associations among study variables.

Despite these weaknesses, this study may provide some valuable insights into designing school activities or programs for academic procrastination in Chinese college students. First, positive peer relationships can facilitate better academic performance in college students. As such, cooperative peer activities and group learning with definite purposes are recommended to be organized regularly. For students with insecure attachment relationships with peers, they will need assistance in developing supportive relationships with their peers at college, thereby aiding them in academic performance (Holt et al., 2018). Second, these activities may highlight the positive role of future time perspective and grit. For instance, school activities should be designed to alter time perspectives, so that students with a present orientation may learn how to think in a more future-oriented manner (Mello and Worrell, 2015), and to regard future orientation as more confident, optimistic, and expansive. Moreover, when confronting challenges and setbacks, undergraduate students should prioritize long-term goals, and goals-related practice should be made to improve grit (Lan and Moscardino, 2019a).

## Conclusion

This study contributes to our understanding of the mediating and moderating processes in the association between peer attachment and academic procrastination in undergraduate students. Based on an exploratory approach, we examine a moderated mediation model emphasizing the role of future time perspective and grit in such an association. These findings suggest that interventions targeting the enhancement of positive peer relationships and personal characteristics (e.g., grit) may prevent or reduce academic procrastination; moreover, future time perspective is integral to human motivation, and college students who regard future orientation as pessimistic should be paid specific attention by university-based counseling services.

## Data Availability Statement

The datasets generated for this study are available on request to the corresponding author.

## Ethics Statement

The studies involving human participants were reviewed and approved by Northwest Minzu University. The patients/participants provided their written informed consent to participate in this study.

## Author Contributions

HJ conceived and drafted the manuscript. WW helped with data collection and preparation of the manuscript. XL performed the statistical analyses and critically revised the manuscript. All authors read and approved the final draft of the manuscript.

## Conflict of Interest

The authors declare that the research was conducted in the absence of any commercial or financial relationships that could be construed as a potential conflict of interest.

## Footnotes

1 As indicated by prior research (Baron and Kenny, 1986, p. 1174), moderator variables always function as independent variables. That is, a moderator (i.e., future time perspective) and an independent variable (peer attachment) are at the same level in regard to their roles as antecedents of academic procrastination in the current study.

2 The duration of data collection process in this study was due to two constraints: (a) we had difficulties negotiating the procedures with responsible principals of the corresponding universities. To achieve a desirable sample size, we took a relatively long process to find suitable sampling locations; and (b) the principal investigator suspended this project because of research leave.

3 Instead of examining the isolated effects of single variables, it may be more informative to investigate how multiple factors (across domains or contexts) jointly or interactively shape individuals’ functions (Zhou et al., 2012). When interaction effects are present, it means that interpretation of the single variables (i.e., main effects) may be incomplete or misleading (Aiken and West, 1991). Despite seemingly counterintuitive, a moderator effect within a correlational framework can change the direction of the correlation (Baron and Kenny, 1986, p. 1174). That is, when a moderator is added into the linear regression model, the original direction of the main effects can be changed. Such an effect has occurred in the current study; in this case, we only interpret the significant interaction term instead of explaining these main effects (Aiken and West, 1991).
